# Development of a lambda Red based system for gene deletion in *Chlamydia*

**DOI:** 10.1371/journal.pone.0311630

**Published:** 2024-11-14

**Authors:** Yibing Wang, Robert Suchland, Amy Hua, Steven Carrell, Daniel Rockey, Kevin Hybiske

**Affiliations:** 1 Department of Medicine, University of Washington, Seattle, Washington, United States of America; 2 Department of Biomedical Sciences, Oregon State University, Corvallis, Oregon, United States of America; University of Texas Health Science Center at San Antonio, UNITED STATES OF AMERICA

## Abstract

The ability to efficiently target loci in the *Chlamydia trachomatis* genome for deletion remains a desirable goal in the field and new strategies need to be developed and refined. Here we describe the development and application of a lambda red recombineering system for *Chlamydia*. Using a non-replicative plasmid encoding key lambda Red components and targeting sequences, we demonstrate the efficient deletion of numerous gene targets in the model chlamydial strains *C*. *trachomatis* L2/434 and *C*. *muridarum*. For initial development of the system, we targeted the *incA* gene of *C*. *trachomatis* for deletion. Deletion mutants containing a chloramphenicol resistance marker were recovered within 3 rounds of selection, or 2 rounds of passaging, in McCoy cells and the resulting clones (CTΔ*incA*) were verified by PCR-based genotyping and whole genome sequencing. Phenotypic assessment of host cells infected with CTΔ*incA* was performed by immunofluorescence microscopy and confirmed the lack of IncA expression and the uniform presence of nonfusogenic vacuoles (inclusions) across CTΔ*incA*-infected monolayers. To explore the utility of this system, we deleted 5 additional candidate virulence factors in *C*. *trachomatis* and *C*. *muridarum*, including deletions of single and multiple genes. We expect lambda Red recombineering to offer a powerful new strategy for making gene deletion and/or replacement mutants in *Chlamydia*.

## Introduction

Infections caused by the obligate intracellular bacterium *Chlamydia trachomatis* are the most commonly reported bacterial infection in the United States, and are responsible for numerous genital tract, rectal, and ocular pathologies in humans [1–4]. Many fundamental aspects of chlamydial pathogenesis and infection remain unresolved due to historical barriers in genetic engineering of *Chlamydia* [5, 6]. The past decade has witnessed a series of notable advances in *Chlamydia* genetics. Spearheaded by the discovery of how to stably transform *C*. *trachomatis* with a shuttle vector [7], the field has made key advances including inducible gene expression systems [8, 9], targeted gene inactivation by mobile introns and allelic exchange [10, 11], transposon mutagenesis [12, 13], and gene repression by CRISPRi [14]. Several of these technologies have enabled the generation of knockout mutant strains, although none have progressed to the advanced stage of genome-wide library generation due to ongoing challenges with efficiency and throughput. Notably, the natural competency of *Chlamydia* for homologous recombination in vitro and in vivo [11, 15–17] highlights the versatility and potential of allelic exchange as a strategy for efficient gene-targeted mutagenesis in *Chlamydia*.

Recombineering of bacterial genomes by the lambda Red system has been widely used for numerous bacterial systems [18–25], including for the purpose of engineering complete libraries of gene knockout clone [26]. This engineering platform consists of expressing the three lambda (λ) phage proteins Beta, Gam, and Exo. These proteins encode for a single-stranded DNA (ssDNA) binding protein, a nuclease suppressor, and a 5’-3’ exonuclease, respectively. Upon introduction of recombinant double-stranded DNA (dsDNA) with flanking sequences homologous to a chromosomal target site into a bacterial host, donor DNA is processed by lambda Red proteins into fragments with single-stranded overhangs and replaced into the target site by homologous recombination. For many bacterial systems, the introduction of linear dsDNA is preferred; however, plasmid-derived dsDNA has also been used [27].

In this study we report the development of a lambda Red-mediated system for gene deletion in *C*. *trachomatis*. Our approach uses a non-replicative plasmid encoding lambda Red components and targeting sequences to deliver dsDNA into *C*. *trachomatis* and achieve gene replacement by homologous recombination. We show that knockout mutants can be accomplished reproducibly, and we further demonstrate the versatility of the system by applying it to the related model organism *C*. *muridarum*. Altogether we report the generation of six single- and multi-gene deletions in candidate *Chlamydia* virulence factors. The applicability and potential of this new system lies in the ability to disrupt candidate genes for downstream functional investigation of associated phenotypes.

## Materials and methods

### Biological resources

Propagation of *C*. *trachomatis* and *C*. *muridarum* was performed in McCoy cells (ATCC # CRL-1696) cultured in full growth medium (FGM) with Dulbecco’s modified Eagles’ medium (DMEM, Gibco^™^ DMEM, High Glucose, GlutaMAX, Fisher Scientific, Cat# 10-566-024) supplemented with 10% fetal bovine serum (FBS) in a 37°C 5% CO_2_ incubator.

### Reagents and enzymes

In-Fusion Snap Assembly (Takara Bio, Cat# 638945) was used for assembling plasmids from PCR fragments. PCR reactions were carried out by using either Q5 High-Fidelity DNA Polymerase (NEB Cat # M0491S) or Phusion^®^ High-Fidelity DNA Polymerase (NEB Cat # M0530S). Specific antibodies to L2 MOMP (1C2), IncA (3H7), and CM MOMP (33b) were provided by Robert Suchland, Dan Rockey, and Harlan Caldwell, respectively. Oligonucleotides were purchased from Integrated DNA Technologies.

### Construction of pLRED vectors

The intermediate cloning plasmid pLRED was constructed via In-Fusion mediated assembly of three PCR fragments amplified from p46Cpf1-OP2 [28], pSW2-GFPCAT [7], and pL2-tet-IncA-APEX2 [29]. Assembly was performed in accordance with the manufacturer’s protocol. This plasmid contains the lambda Red genes *gam*, *beta*, and *exo* under the control of a tetracycline-inducible promoter (S1 Fig) [8, 29]. The pLRED cloning plasmid was used as source sequences for the streamlined subsequent building of target-specific vectors.

**Fig 1:**
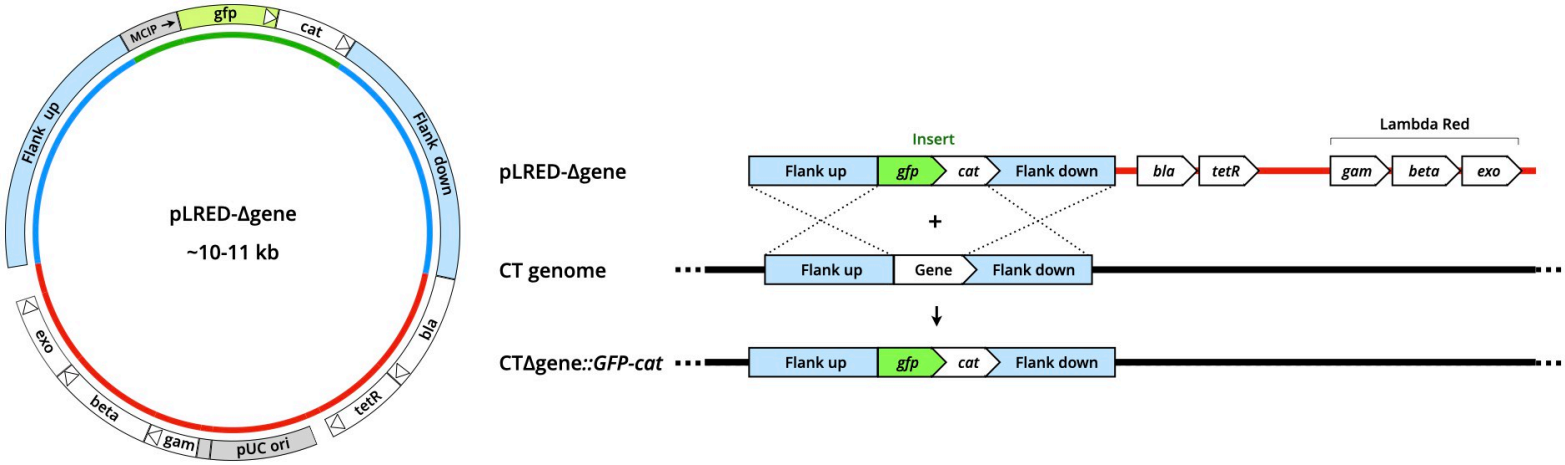
Lambda Red mediated gene deletion in *Chlamydia*. (Left) Circular map of the universal gene-targeting vector pLRED-Δgene displays key features: insertion cassette containing *gfp* and *cat* (green), *C*. *trachomatis* genomic DNA upstream of the target gene (blue), genomic DNA downstream of the target gene (blue), and vector backbone (red) containing sequences for propagation and selection in *E*. *coli* and the lambda Red genes *gam*, *beta*, and *exo* under regulation of a tetracycline-inducible promoter. (Right) Linear DNA schematic of target gene deletion in *C*. *trachomatis* (CT) by pLREDΔgene and homologous recombination.

For construction of target-specific vectors, four PCR fragments were prepared and assembled by In-Fusion cloning: (i) vector backbone containing *gam*, *beta*, *exo*, pUC *ori*, *bla*, and *tetR*; (ii) selection insert containing *gfp* and *cat*; (iii) flanking sequences upstream of the targeted gene; (iv) flanking sequences downstream of the targeted gene. Genomic DNA from *C*. *trachomatis* L2 was used as source DNA for the amplification of flanking sequences. The arrangement of these fragments into a target-specific vector is illustrated in Fig 1. PCR reactions were performed using either Q5 High-Fidelity DNA Polymerase or Phusion High-Fidelity DNA Polymerase, and the resulting fragments were purified (NucleoSpin Gel and PCR Clean-Up kit, Takara Cat # 740609) from agarose gels prior to cloning. Antibiotic resistant (amp^R^ and cam^R^) *E*. *coli* clones were picked and grown for plasmid DNA extraction (QIAprep Spin Miniprep kit, Qiagen Cat # 27104). Sequence verified plasmids were finally transformed into *dam*^–^*/dcm*^*−*^competent *E*. *coli* for extraction of plasmids to be used in *Chlamydia* transformations.

### Generation of *C*. *trachomatis* and *C*. *muridarum* deletion mutants

*C*. *trachomatis* L2 and *C*. *muridarum* stocks for transformation were grown from two 6-well plates of McCoy cells (50–100% infection) in FGM supplemented with 1 μg/ml cycloheximide. *Chlamydia*-infected cells were collected by centrifugation at 3500 × g for 10 min. Pellets were suspended in 1 ml of 10% PBS in water and transferred to a bijoux tube with glass beads and vortexed at high speed for 1 min to break open the cells and release the EBs. Cell debris was removed by centrifugation at 250 × g for 5 min and the supernatant containing partially purified EBs was mixed with an equal volume of 4SP solution (phosphate/sucrose buffer, 16 mM Na_2_HPO_4_ pH 7.1 and 0.4 M sucrose) and was stored at -80°C. Small aliquots were set aside for transformation experiments.

Procedures for transforming both *Chlamydia* species are similar to those described in Wang et al [7] with some modifications. For selection of *Chlamydia* transformants and gene deletion mutants, FGM was supplemented with 1 μg/ml cycloheximide, 0.5 μg/ml chloramphenicol, and 10 ng/ml anhydrous tetracycline (ATc). Typically for one transformation experiment, 5–10 μl *Chlamydia* EBs (10^9^ IFU/ml) and 10–20 μg plasmid DNA (concentration >300 ng/μl) were mixed in a 1.5 ml microtube and an equal volume of 2× concentrated CaCl_2_/tris buffer was added (final concentration of 10 mM Tris pH 7.4 and 50 mM CaCl_2_) for 10–20 min. Meanwhile, approximately 5 × 10^6^ McCoy cells from a T75 flask at 70–80% confluence were collected and washed with phosphate buffered saline (PBS) before resuspension in CaCl_2_/tris buffer. Half of the McCoy cells were transferred into a bijoux tube and the EB/plasmid mixture was combined with cells and incubated for about 10 min at room temperature with occasional mixing. Suspensions were fed with 2.8 ml FGM, and about 0.5 ml was aliquoted into each well of a 6-well plate containing 2 ml FGM, and allowed to settle for 1–3 h in a 37°C CO_2_ incubator. Plates were centrifuged at 700 × g for 30–60 min, changed with 2 ml fresh FGM supplemented with 1 μg/ml cycloheximide, 0.5 μg/ml chloramphenicol, and 10 ng/ml ATc and incubated at 37°C 5% CO_2_ for 2–3 days. In some instances, chloramphenicol selection was initiated on the following day. This stage of the procedure was designated as T0 (Transformed plate 0). Conditions were adjusted as necessary to produce infection levels of ~50–60% of host cells.

At 2–3 days post-transformation, bacteria were subsequently passaged by bead lysis as described above followed by infection of fresh McCoy cells in 6-well plates under chloramphenicol selection and ATc induction. Passage numbers were assigned sequentially, for example the passage of T0 cultures was designated as P1, and the resulting infection plate was designated as T1. Generally, only a portion of T0 (⅙-⅓) was used to infect the P1 6-well plate of McCoy cells using a 1:2 series dilution scheme. The T1 plates were typically allowed to incubate for 2–7 days post-infection and monitored visually each day for the emergence of green and resistant inclusions. At this time, *Chlamydia* mutants were passaged another time (P2) onto fresh McCoy cells (T2), and incubated for 2–4 days. We determined that careful management of infectious titers of each passage could reliably produce wells with low numbers of plaque-like clusters, which subsequently facilitated detection and clonal propagation by plaque picking. If necessary, cultures were propagated for additional round(s).

### PCR analysis of mutants

Genomic DNA from *Chlamydia* deletion mutants and parental *Chlamydia* strains were extracted using Macherey-Nagel NucleoSpin Tissue (Takara, Cat # 740952.50). The gDNA samples were diluted to 10 ng/μl and ~1 μl DNA was used as PCR template in 25 μl PCR reactions. For plasmid control, ~0.5 ng of plasmid was used. To verify the insertion of recombinant inserts and deletion of target genes, PCR was performed using primers specific to regions outside the boundaries of flanking regions. To verify the existence and position of recombination ends, ‘outside’ genomic primers were used in combination with primers specific to sequences at the ends of the recombinant insert. All oligonucleotide primers are summarized in S1 Table. Q5 High-Fidelity DNA Polymerase or Phusion^®^ High-Fidelity DNA Polymerase was used to amplify PCR products, and the PCR reactions annealing temperatures were determined by using NEB Tm Calculator. PCR products were analyzed by gel electrophoresis and sequences were verified by Sanger sequencing.

### Whole genome sequencing and analysis

The Qiagen blood and tissue kit was used to produce genomic DNA from purified EBs for each of six recombinants. The purified DNA was the pooled and submitted to the University of Oregon Genomics and Cell Characterization Core Facility for sequencing on a PacBio Sequell II. Reads were acquired and sorted based on the presence of *cat*. This pool was then either analyzed directly for presence on the genome or sorted again based on short sequences unique to each lambda red recombinant. Finally, the number of *bla* positive/*cat* positive reads were identified to assess the presence of single crossover events. All subsequent analysis was conducted in the Geneious sequence analysis suite.

### Immunofluorescence microscopy

McCoy cells were grown on coverslips in 12 mm^2^ shell vials and were infected with either L2 wt, CMwt, L2ΔincA, or CMΔincA at a MOI>1.0 using standard chlamydia culture methods [13, 15]. L2 and CM preps were fixed with methanol at 28 and 20 hours post infection (hpi), respectively, to accommodate growth rate differences. Infected L2 coverslips were labeled with anti-L2 MOMP and anti-IncA monoclonal antibodies. CM coverslips were labeled with an anti-CM MOMP antibody (gift from Harlan Caldwell) and counterstained with Evans Blue to help distinguish inclusion morphology. Appropriate secondary antibodies were used and coverslips were mounted on glass slides using Vectasheld mounting media (Vector Laboratories) containing the DNA-specific dye 4′,6-diamidino-2-phenylindole (DAPI). Images were acquired under oil immersion on a Nikon Eclipse 80i microscope using Nikon NIS elements software.

## Results

### Vector design

The goal of this study was to develop a straightforward system for targeted gene deletion and replacement in *Chlamydia*. We rationalized that such a system would (i) leverage DNA vector construction that could be assembled in 1–2 steps, (ii) contain elements for facilitating visual identification and selecting recombinant *Chlamydia* clones, and (iii) allow gene deletion in multiple *Chlamydia* species or strains. We turned to the lambda Red recombineering system due to its broad efficacy for *E*. *coli* and other bacterial systems. We ultimately settled on a double stranded (ds) DNA plasmid-based approach for delivery of lambda Red and recombinant DNA elements, after unsuccessful attempts to generate recombinant *Chlamydia* using either linear dsDNA or dual plasmid strategies. The constructed vector includes four key components: a vector backbone containing the lambda Red gene cassette under regulation of tetracycline-inducible promoter, an insertion cassette containing green fluorescence protein (*gfp*) and chloramphenicol acetyltransferase (*cat*), and flanking sequences (typically ~2 kb) immediately upstream and downstream of the gene to be deleted or replaced (Fig 1). We describe these DNA vectors as pLRED-Δgene wherein ‘gene’ is replaced with the specific gene(s) that is targeted by a particular vector. This vector does not contain sequences from any known *Chlamydia* native plasmids (i.e., no chlamydial replication of origin) and thus does not propagate as an episome in *C*. *trachomatis*.

### Deletion of *incA* in *C*. *trachomatis* L2/434/Bu

For development and proof of concept, we targeted *incA* in *C*. *trachomatis* L2 because of the documented capacity of this gene to tolerate mutations in this strain background and also the distinctive morphological phenotype of *incA* deficient strains that is amenable to real-time visual evaluation [10, 30]. We settled on a simplified procedure that could be reproducibly followed for additional gene targets and *Chlamydia* organisms, and thus could be easily adopted by other research groups. We constructed the vector pLRED-ΔincA through In-Fusion cloning and incorporation of ~2 kb homologous flanking sequences immediately upstream and downstream of *incA*. We transformed *C*. *trachomatis* with pLRED-ΔincA in a standard McCoy cell infection system, under sustained chloramphenicol selection and anhydrotetracycline hydrochloride (ATc) induction of the lambda RED cassette [8]. CTΔ*incA*::*gfp-cat* transformants (simplified as CTΔ*incA*) were recovered within a total of 10 days, during which 2–3 rounds of chloramphenicol selection and green fluorescence monitoring were conducted. Within this timeframe, numerous plaque-like clusters of infected cells were found that displayed the characteristic Δ*incA* phenotype of numerous unfused inclusions in individually infected cells.

**Fig 2:**
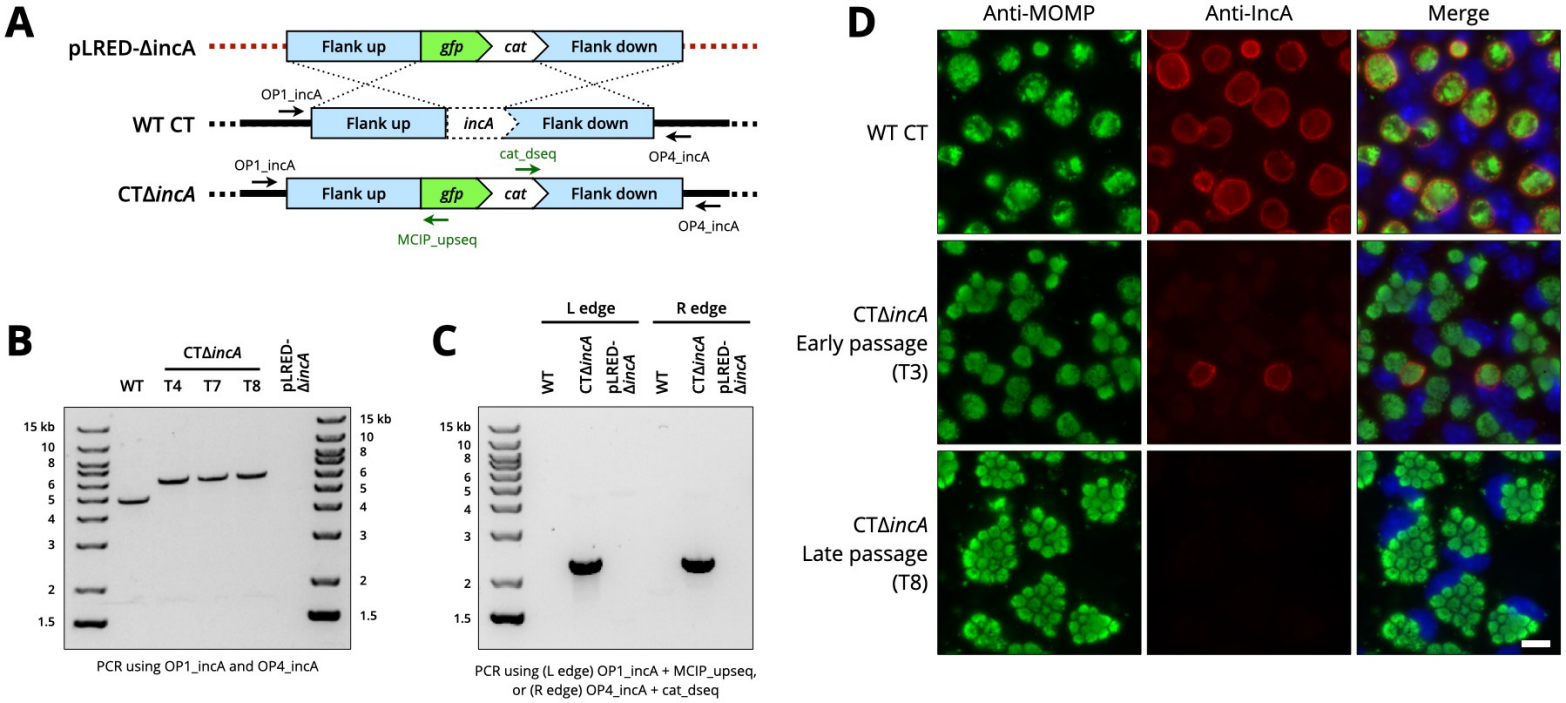
Generation of CTΔ*incA* deletion mutants. (A) Linear DNA schematic of recombinant insert, the *incA* locus in WT *C*. *trachomatis*, and the deletion locus in CTΔ*incA*. (B) PCR analysis of WT, a temporal progression of CTΔ*incA* isolates, and the control plasmid pLRED-Δ*incA*, using the chromosomal primers OP1_incA and OP4_incA situated outside the flanking regions. (C) PCR analysis of the same DNA samples using combinations of chromosomal and insert primer pairs that bridge the left and right recombination edges. (D) Phenotypic analysis of WT CT, and early (T3) and late (T8) isolates of CTΔ*incA*. McCoy cells were infected with the indicated strains for 28 h and analyzed by immunofluorescence microscopy with specific antibodies to CT MOMP (green) and IncA (red), and counterstained with DAPI (blue). Scale bar, 20 μm.

To confirm the deletion of *incA* from *C*. *trachomatis*, genomic DNA (gDNA) was extracted from several of these isolates at progressive stages of selection and subjected to PCR-based genotyping analysis. We used three approaches to confirm the replacement of *incA* with the recombinant *gfp-cat* DNA insert. First, we amplified PCR fragments from both wildtype and transformed *C*. *trachomatis* isolates using oligonucleotide primers specific to chromosomal regions outside the flanking sequences (Fig 2A). DNA from wildtype *C*. *trachomatis* yielded a single band at the predicted size of ~5 kb (Fig 2B). DNA from all transformed isolates produced individual bands at ~6 kb, consistent with the larger size of the *gfp-cat* cassette compared to *incA* (Fig 2B). The pLRED-ΔincA vector was used as negative control and yielded no amplified product (Fig 2B). These results demonstrate that the chromosomal *incA* gene was replaced with the larger *gfp-cat* insert at a time prior to the T4 isolate, and this genotype was maintained throughout serial growth to the T8 isolate. As a second approach, we performed PCR with primer pairs that bridged each of the recombination ends—one of the chromosomal primers used above in combination with a primer inside the *gfp-cat* insert (Fig 2A). PCR analysis with these primer pairs yielded amplicons only for gDNA from transformed isolates, with no detectable bands for gDNA from wildtype *C*. *trachomatis* or plasmid DNA (Fig 2C). As a final approach, we performed long-read whole genome sequencing of gDNA extracted from the mutant and the expected replacement of *incA* with *gfp-cat* was confirmed by analysis of approximately 1500 read sequences covering the *incA* locus (S2 Fig). These combined results demonstrate that recombination had occurred at the expected chromosomal location, and the deletion of *incA* from CT strains.

We next conducted phenotypic analysis of CTΔ*incA* clones to validate the absence of IncA protein expression and the presence of non-fusogenic inclusions which are diagnostic of *C*. *trachomatis* IncA deficient mutant strains [10, 30]. We infected McCoy cells with Δ*incA* and WT CT L2 at a MOI >1 and subjected them to immunofluorescence microscopy analysis at 28 hpi. Cells infected with WT CT L2 harbored single large inclusions that were positive for IncA expression on their inclusion membranes (Fig 2D). An early passage (T3) isolate of CTΔ*incA* resulted in many cells that contained multiple, non-fused inclusions per cell; the inclusions in these cells were devoid of IncA protein expression (Fig 2D). Because the early passage isolate was not yet clonal for the CTΔ*incA* mutant, infected cells with WT morphology and IncA expression were present in a minority of cells (Fig 2D). Cells infected with late passage isolates were essentially clonal for CTΔ*incA*, as all infected cells contained multiple unfused inclusions that were negative for IncA expression (Fig 2D).

### Deletion of candidate virulence loci in *C*. *trachomatis*

**Fig 3:**
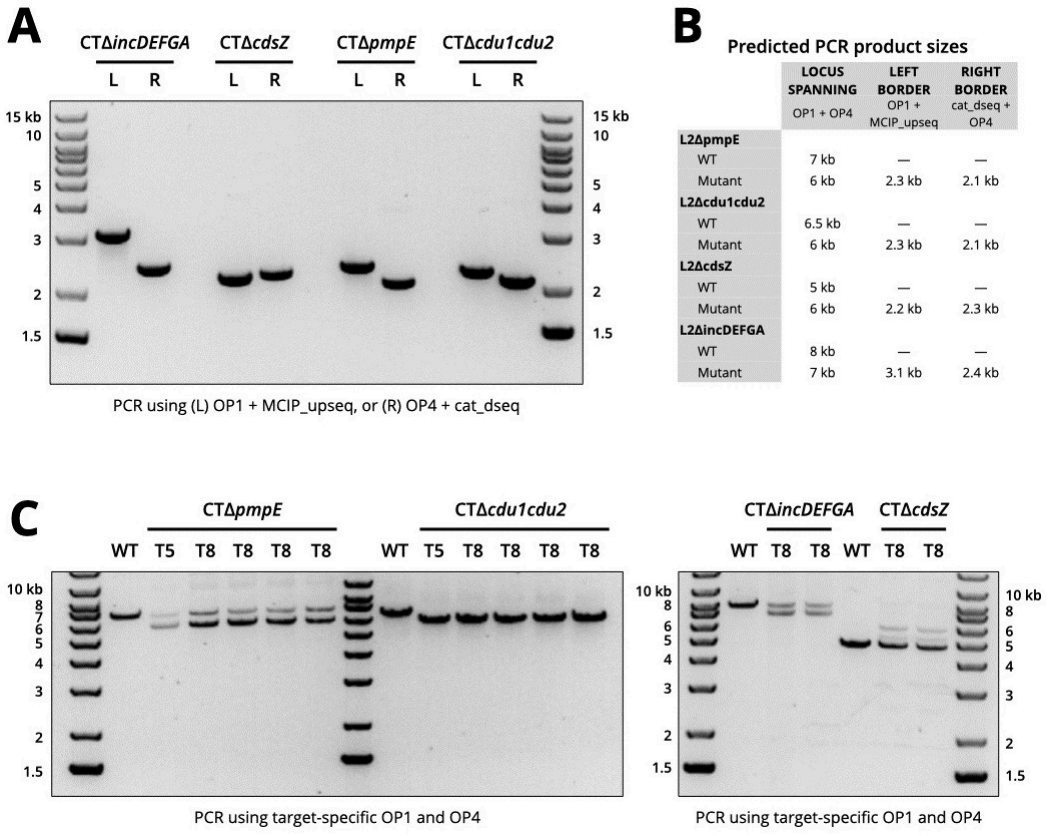
Deletion of additional loci in *C*. *trachomatis*. (A) PCR products from four mutants (gDNA from T5) using a primer on insert (MCIP_upseq_1 or cat_dseq_2) paired with a primer on *C*. *trachomatis* gDNA outside flanking sequences (OP1 or OP4, not on plasmid). (B) Summary table of expected PCR product sizes for each reaction. (C) PCR products from WT and mutant gDNA using locus-specific chromosomal primers (OP1 and OP4) situated outside the homologous flanking regions.

Based on our initial success, we sought to explore the reliability and versatility of the lambda Red system by targeting additional gene targets in *C*. *trachomatis*. First, we made repeated independent attempts to knock out *incA* from *C*. *trachomatis* and succeeded in producing new mutant clones that were indistinguishable from the first CTΔ*incA* mutant (S3 Fig). Next, following the same scheme as described above, we built pLRED vectors targeting: *pmpE*, *cdsZ*, the *cdu1cdu2* gene cluster, and the *incDEFGA* gene cluster. These targets were selected due to their involvement in type III secretion processes and other aspects of host cell manipulation [17, 31–34]. For each transformation attempt, mutants were successfully recovered after several serial passages of chloramphenicol selection. We isolated gDNA from isolates of each mutant strain and performed PCR-based genotyping to determine whether allelic replacements had occurred as designed. We determined whether recombination had occurred at the correct chromosomal location for each mutant by performing PCR with primers spanning the recombination borders on both ends, i.e., chromosomal primer OP1 paired with the upstream insert primer MCIP_upseq, and the chromosomal primer OP4 paired with the downstream insert primer cat_dseq. Products of expected sizes were generated for each reaction and mutant isolate (Fig 3A and 3B), demonstrating that *gfp-cat* inserts had been delivered into each mutant’s chromosome via homologous recombination. No specific PCR products when using WT *C*. *trachomatis* gDNA or plasmids as DNA template. We additionally assessed the replacement of endogenous genes with the *gfp-cat* insert using primers designed against gDNA outside each target’s respective flanking regions (OP1 and OP4) and evaluating the predicted shift in PCR product size due to gene replacement (Fig 3B and 3C). Deletions were achieved for each transformation; however, the time required for each mutant to become clonally pure was variable. For CTΔ*cdu1cdu2*, replacement of the gene pair with *gfp-cat* was prevalent across the mutant population by T5, as evidenced by a predicted shift of that PCR-amplified chromosomal locus from 5 kb to 6 kb (Fig 3C). For CTΔ*pmpE*, CTΔ*cdsZ*, and CTΔ*incDEFGA*, the appropriate PCR products were present by T5 and maintained through T8; however, products corresponding to the intact WT gene(s) were also present (Fig 3C), indicating that further clonal propagation is necessary to derive clones of double crossover recombinants.

### Gene deletion mutants in *C*. *muridarum*

**Fig 4:**
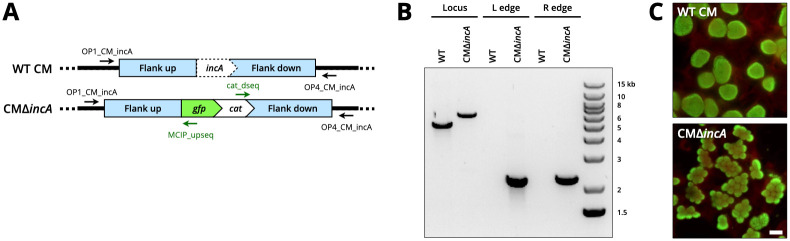
Deletion of *incA* in *C*. *muridarum*. (A) Linear DNA schematic of the *incA* locus in WT *C*. *muridarum* and the deletion locus in CMΔ*incA*. (B) PCR analysis of WT and CMΔ*incA*. “Locus” used chromosomal primers OP1_CM_incA and OP4_CM_incA that recognize sequences outside the flanking regions. “L edge” and “R edge” PCR reactions used combinations of chromosomal and insert primers that bridge the left and right recombination edges, respectively, as indicated in panel A. (C) Phenotypic fluorescence microscopy analysis of McCoy cells infected with WT *C*. *muridarum* or CMΔ*incA* at 20 hpi, using an specific antibody to *C*. *muridarum* MOMP (green) and counterstained with Evans blue (red). Scale bar, 20 μm.

To further investigate the versatility of the lambda Red system, we explored adapting it to the rodent-tropic species *C*. *muridarum*. Advancing genetic tools for this model organism is highly desirable for the purpose of identifying and characterizing chlamydial factors important for in vivo infection. We hypothesized that the universal backbone design of the pLRED system would facilitate an easy translation of the tool to *C*. *muridarum*. We assembled the vector pLRED-Δ*CM_incA* with flanking sequences homologous to chromosome regions upstream and downstream of the *incA* gene in *C*. *muridarum* (Fig 4A), and transformed *C*. *muridarum* in McCoy cells supplemented with ATc and chloramphenicol. Resistant mutants exhibiting green fluorescence were visually identified by passage 2 (7–10 days total), and selection of plaque-like clusters of mutants was performed to produce clonal isolates. After plaque purification, we isolated gDNA from several mutant clones and performed PCR analysis to confirm the Δ*incA*::*gfp-cat* genotype. Using primers located outside the *incA* locus in *C*. *muridarum* and homologous flanking regions, single PCR products of expected and differing sizes were obtained from WT *C*. *muridarum* and CMΔ*incA* gDNA (Fig 4B). Using primers spanning each of the recombination ends we also obtained PCR products of the expected sizes (Fig 4B), indicating the replacement of *incA* with the *gfp-cat* recombinant insert. During selection and propagation of CMΔ*incA* we observed the presence of infected cells that contained multiple chlamydial inclusions, suggesting that the nonfusogenic inclusion defect characteristic of *incA*^*null*^ *C*. *trachomatis* strains was similar in *C*. *muridarum*. We investigated this further by performing immunofluorescence on McCoy cells infected with high titers (MOI>1) of either WT *C*. *muridarum* or CMΔ*incA*, and labeling cells at 20 hpi with a specific antibody to *C*. *muridarum* MOMP. Whereas cells infected with WT *C*. *muridarum* uniformly contained large single inclusions, that reflect the outcome of IncA-mediated fusion of parent inclusions, cells infected with CMΔ*incA* were filled with multiple small inclusions (Fig 4C). These results are consistent with the phenotype of *incA* deficient *C*. *trachomatis*.

## Discussion

The ability to create gene deletion mutants in a bacterial pathogen is a core requirement for the experimental identification and characterization of candidate virulence factors for their functional roles in pathogenesis. This has historically not been possible for *Chlamydia* due to a lack of modern genetic systems. Important new tools have emerged over the past decade, all of which were enabled by the initial development of a plasmid transformation system for *C*. *trachomatis* [7]. Highlighted progress in genetic tool development includes transposon and chemical mutagenesis for making random gene disruptions [12, 13, 35–37], and Targetron and Fluorescence-Reported Allelic Exchange Mutagenesis (FRAEM) technologies for making targeted allelic exchange mutants [10, 11]. Notably, FRAEM leverages the endogenous capability of *C*. *trachomatis* for homologous recombination, highlighting the effectiveness of recombination based strategies as a platform for genetic tool development in *Chlamydia*. While effective, these strategies have not progressed to genome-scale throughput, and the field would benefit from having additional targeted gene disruption technologies so that researchers can choose the optimal approach for their particular needs.

We have developed a reproducible and versatile system for creating gene knockouts in *Chlamydia*. We found that transient expression of the lambda Red genes in *Chlamydia*, delivered through a vector that cannot propagate in *Chlamydia*, produced double crossover recombinant strains. Furthermore, we show that the lambda Red recombineering system has broad utility for *Chlamydia*, by using the same vector backbone construct to generate target-specific vectors and gene knockout mutants in both *C*. *trachomatis* L2/434 and *C*. *muridarum*. These are the most widely used strains for in vitro and in vivo *Chlamydia* research, in particular for investigations on chlamydial pathogenesis, and thus the lambda Red system has the potential for wide adoption by other groups. For the initial development of the system in both of these genome backgrounds, we selected the *incA* gene for deletion. This decision was driven by the established capacity of this gene to tolerate disruptions [10, 30], and the distinctive morphological phenotype of host cells infected with *incA* mutants of *Chlamydia*. Clones of Δ*incA* mutants for *C*. *trachomatis* and *C*. *muridarum* were obtained within approximately 4 rounds of propagation under sustained chloramphenicol selection, after which no presence of the wildtype allele remained. As a ‘real world’ test of the system versatility and reliability, we next used it to target 4 additional loci in *C*. *trachomatis* that have not been previously targeted by existing technologies. Using the same workflow developed during our proof of concept stage, we were successfully able to generate deletion mutants for each target.

Our initial attempts to adapt the lambda Red system for *C*. *trachomatis* used a stable shuttle vector containing the lambda Red genes under inducible regulation, and linear, PCR-amplified dsDNA as donor DNA containing flanking sequences and a selection cassette. These efforts were not successful, and we experienced similar failures after incorporating Chi sites into our DNA. As for other bacteria systems, we suspect that RecA and/or the RecBCD complex may interfere with the stability of linear DNA in *Chlamydia* and thus recombination from linear dsDNA sequences. Further work needs to be done to explore this possibility and eventually overcome this experimental barrier. We also attempted a version of the lambda Red system wherein the lambda Red genes and targeting sequences were supplied on separate shuttle vectors. *C*. *trachomatis* transformed sequentially with these vectors failed to produce recombinants in our testing. Ultimately, the optimal approach proved to be the single vector, pLRed system described in this study.

We demonstrate that the lambda Red system can be exploited to delete clusters of two or more genes in *Chlamydia*. This technical capability allows for adjacent genes with potentially similar or redundant functions to be examined for phenotypic defects. We targeted the putative deubiquitinase genes *cdu1* and *cdu2* because of the compelling yet modest phenotypes associated with a *cdu1* transposon mutant [32], the lack of knowledge regarding the function of Cdu2, and the potential value of possessing a more complete deubiquitinase deficient *cdu1cdu2* mutant for functional investigation. We also targeted the *incDEFGA* cluster that encodes five inclusion membrane proteins; several of these genes have established roles in *C*. *trachomatis* pathogenesis [34], and whether this genomic locus is required for optimal infection of host cells is an open question. For CTΔ*cdu1cdu2*, a clonal population containing the deletion emerged quickly, prior to isolate T5. For CTΔ*incDEFGA*, we observed the persistence of the wildtype locus along with the deleted locus, out to isolate T8. Efforts to separate these recombinant strains by extensive plaque cloning are ongoing. One possible explanation for these observations is that mutants with pronounced fitness defects may fail to outgrow any wildtype organisms that are incompletely removed during selection; support for this possibility comes from the observation that wildtype alleles remain in the mutant population after many rounds of selection. It is also possible that single cross recombinant strains containing the plasmid inserted in their chromosomes may exist as part of the mutant pool. In these strains, recombinant plasmid DNA may transition between free plasmid and chromosomal insertion, similar to what has been observed for *C*. *trachomatis* [38] and *C*. *suis* [39]. Independent of the possible presence of these strain genotypes, double crossover mediated deletion of targeted alleles occurred readily, as the genome sequencing results for CTΔ*incA* indicates, and it will be necessary to extensively clone and expand some mutant pools to derive stocks ready for further experimentation.

This work raises numerous opportunities for expansion and further development. We demonstrate that the system is equally effective in *C*. *trachomatis* L2 and *C*. *muridarum*; however, further work should attempt to adapt the method to additional *Chlamydia* species and strains such as model urogenital *C*. *trachomatis* strains and *C*. *pneumoniae*. It will also be useful to optimize the system for efficient insertion of recombinant DNA into the *Chlamydia* chromosome, as opposed to gene deletion. At present, the available tools for expressing genes with point mutations or protein tags are limited to plasmid-based expression following transformation of *Chlamydia* with a shuttle vector. Calibration of the expression of plasmid-encoded recombinant genes to chromosomal counterparts, even if endogenous promoters are used, is very challenging. Efforts should also be made to develop the system for marker removal from recombinant chromosomes. As a proof of concept, we generated an additional CTΔ*incA* mutant with FRT sites flanking the *GFPcat* insert, analogous to what has been demonstrated for marker removal in *C*. *trachomatis* using Cre-lox [40]. Finally, the mechanism of lambda Red mediated recombination in *Chlamydia* should be explored further, in particular for determining the minimal length of DNA donor homology necessary to efficiently produce recombination events, and also to more exhaustively evaluate methods for using linear dsDNA sequences.

## Supporting information

S1 Table List of oligonucleotide primer sequences. (PDF)

S1 Fig Detailed map and sequence of vector pLRED. (PDF)

S2 Fig Long-read sequencing alignment of the CTΔincA mutant. Read map alignment of ~1500 long-read sequences mapped onto the reference genome of *C*. *trachomatis* L2/434, with ~95% exhibiting a Q-score of at least Q30 (1:1000 base call error). Each track represents an individual sequencing read, with gaps in alignment suggesting possible structural variations or sequencing artifacts. Highlighted in green are CAT markers. The consistent mapping across the orange highlighted region containing the deleted *incA* allele suggests a stable recombination event at this locus.
(TIF)

S3 Fig PCR analysis of additional CTΔincA mutant clones. gDNA was isolated from WT *C*. *trachomatis* L2 and varying temporal isolates (T1-T8) of the two clones indicated and analyzed by PCR using primers located outside the upstream and downstream ~2 kb flanking sequences. Lower weight bands containing the WT *incA* gene were abundant only in gDNA from WT *C*. *trachomatis*. Higher weight amplicons containing the larger *gfp-cat* insert were present only in mutant gDNA, and were detected from all isolates.
(TIF)

S1 Raw images Raw gel images associated with this study. (PDF)
