# Supplementary material for: Development of a lambda Red based system for gene deletion in Chlamydia
Source: PLoS One. 2024 Nov 14;19(11):e0311630. doi: 10.1371/journal.pone.0311630 (PMC11563418; doi:10.1371/journal.pone.0311630)
Supplement: S1 Table — (PDF) [file pone.0311630.s001.pdf]

S1 Table. Primer list

Oligo name	Sequence (5'–3')
Vector_F	TTTCAGGTGGCACTTTTCGGGGAAATG
Vector_R	CATGGATTCTTCGTCTGTTTCTACTGGTATTGGC
Insert_F	ATTAACAAAAATCAGGACAAGGCGG
Insert_R	GTCGACATTAATCTAGATATCGAGCTCG
Ins_upseq	ACACACGCGGCACTTTATAG
Ins_dseq	GCGATTCAGGTTCATCATG
P1_incA	GCGCCTCACTTTCTTTCTTCTCGATACTCTC
P2_incA	CATCAAAGAAGGTTGAGGTACGCGATTGG
P3_incA	TAGTCTTAGGAGCTTTTTGCAATGCAAAACATAACACC
P4_incA	GAAGTGAGGTTTATGACGAAGGTTTATGCGC
OP1_incA	GTATCCTCTGAAACAGTATCCCC
OP4_incA	GGTAGTTTTTGCGTCCGAAAC
P1_incDA	TGCGCTGGGATGAAGAAAAAG
P2_incDA	CTCACTTATAAAACAATGTTTCGGACGC
P3_incDA	CAAAGAAGGTTGAGGTACGCG
P4_incDA	AGCCTTAACTATACGGAATAACTTAGAACC
OP1_incDA	GGAAGAGGCTGAAGACATG
OP4_incDA	CAGCTATCTCGCCTCTATTTG
P1_cdsZ	GAATACCATAGGCCCTGAACATCCTTTAAAAGCC
P2_cdsZ	GAAATCTTTCCTTACCTTGATATGAGCTTGTAGTGCC
P3_cdsZ	GCAGAAGGCGCAACTACAAAACGTC
P4_cdsZ	GCATGCTTCTCTGGTTGTTGATGTAGATGTTACAG
OP1_cdsZ	TCTTATCAATCCAACTCTAGCTATGG
OP4_cdsZ	GCTTCTTCTTTACGACAGTCTTC
P1_pmpE	CGATAGTTTTCTGGTGTGCAAGTCGGAG
P2_pmpE	CTACTCCTCTTCAACCTTCTGTAATTATCTCAATGGGG
P3_pmpE	CATAGTTAAAAGACCAGAGCTCCTCCTGCATTG
P4_pmpE	CACGCAGACACCTTATACACTATCCCTTGG
OP1_pmpE	ATAACGAATGGGAATACACTAGAGG
OP4_pmpE	GATGCGAACTTATTAGGGATGC
P1_cdu1cdu2	GTAAGATTCAACAGGCAGAGTCGCACC
P2_cdu1cdu2	GCTGGCCAACTACGGATTAGAGAACAAATC
P3_cdu1cdu2	GTTGAGTTGGTGGGAGATAACATAGGGC
P4_cdu1cdu2	CCAGTACGTCAGCTAATCCTCCTAGAAGAAATG
OP1_cdu1cdu2	AGACCGTTTTTGTTGAGTTGC
OP4_cdu1cdu2	GTGACTAATGTAACTAATGCAAATGGTAC
OP1_CM_incA	CAACCGTATCTTCAGAAACAATATCTCC
P1_CM_incA	GGTTTATCTTCCTTCTTCTCCCCTACTTCTTTGACAATC
P2_CM_incA	GAACAGTTTCTATTAATGAGGGCTTATAGAGTGATGAGCTTC
P3_CM_incA	TAAAATATTTGATCCTTCGCTCACGATTAAGGCACTCTTC
P4_CM_incA	CGGAGGAAAGGTTCATGACAAAAATTTGTTCGAC
OP4_CM_incA	GATCTTAACAAATTGAAAAATAAGGGCTTGG
