# Supplementary material for: Development of a lambda Red based system for gene deletion in Chlamydia
Source: PLoS One. 2024 Nov 14;19(11):e0311630. doi: 10.1371/journal.pone.0311630 (PMC11563418; doi:10.1371/journal.pone.0311630)
Supplement: S1 Fig — (PDF) [file pone.0311630.s002.pdf]

S1 Figure. Plasmid pLRED features and sequence

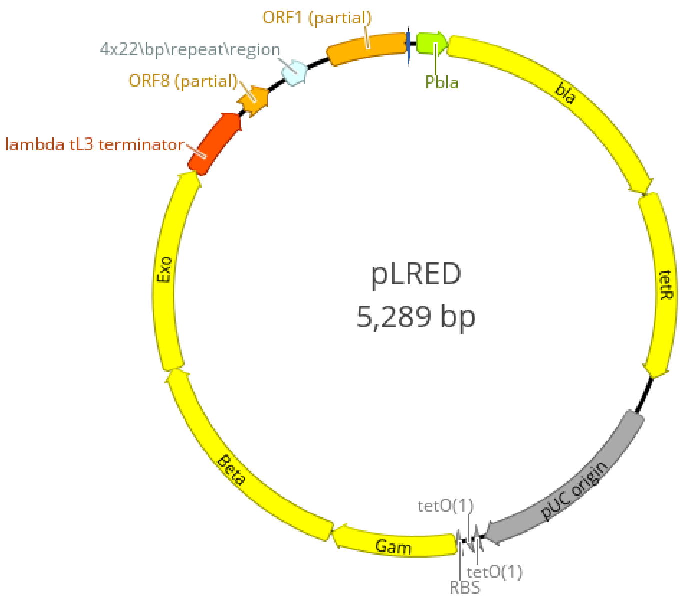

Position on pLRED	Feature	Length	Source (PCR template)
**1→2533**	**Tet-inducible**	**2533 bp**	**pL2-tet-IncA-APEX2**
32→136	bla promoter	105 bp	
137→997	bla	861 bp	
1007→1630	tetR	624 bp	
1757→2430	pUC origin	674 bp	
2451→2469	tetO(1)	19 bp	
2476→2494	tetO(1)	19 bp	
**2534→4683**	**λ RED**		**p46Cpf1-OP2**
2534→2950	gam	417 bp	
2956→3741	beta	786 bp	
3738→4418	exo	681 bp	
4419→4663	λ tL3 terminator	245 bp	
**4684→5289**	**pSW2 replication origin**		**pSW2-GFPCAT**
4684→4787	pSW2 ORF8 (partial)	104 bp	
4857→4944	4x22 bp repeat	88 bp	
5021→5289	pSW2 ORF1 (partial)	269 bp	
**3→4683**	**λ RED vector**	**4681 bp**	**pLRED**

The sequence of pLRED

```
   1 aatttcaggt ggcacttttc ggggaaatgt gcgcggaacc cctatttgtt tatttttcta
  61 aatacattca aatatgtatc cgctcatgag acaataaccc tgataaatgc ttcaataata
 121 ttgaaaaagg aagagtatga gtattcaaca tttccgtgtc gcccttattc ccttttttgc
 181 ggcattttgc cttcctgttt ttgctcaccc agaaacgctg gtgaaagtaa aagatgctga
 241 agatcagttg ggtgcacgag tgggttacat cgaactggat ctcaacagcg gtaagatcct
 301 tgagagtttt cgccccgaag aacgttttcc aatgatgagc acttttaaag ttctgctatg
 361 tggcgcggta ttatcccgta ttgacgccgg gcaagagcaa ctcggtcgcc gcatacacta
 421 ttctcagaat gacttggttg agtactcacc agtcacagaa aagcatctta cggatggcat
 481 gacagtaaga gaattatgca gtgctgccat aaccatgagt gataacactg cggccaactt
 541 acttctgaca acgatcggag gaccgaagga gctaaccgct tttttgcaca acatggggga
 601 tcatgtaact cgccttgatc gttgggaacc ggagctgaat gaagccatac caaacgacga
 661 gcgtgacacc acgatgcctg tagcaatggc aacaacgttg cgcaaactat taactggcga
 721 actacttact ctagcttccc ggcaacaatt gatagactgg atggaggcgg ataaagttgc
 781 aggaccactt ctgcgctcga cccttccggc tggctggttt attgctgata aatctggagc
 841 cggtgagcgt ggctctcgcg gtatcattgc agcactgggg ccagatggta agccctcccg
 901 tatcgtagtt atctacacga cggggagtca ggcaactatg gatgaacgaa atagacagat
 961 cgctgagata ggtgcctcac tgattaagca ttggtaggaa ttaatgatgt ctcgtttaga
1021 taaaagtaaa gtgattaaca gcgcattaga gctgcttaat gaggtcggaa tcgaaggttt
1081 aacaacccgt aaactcgccc agaagctagg tgtagagcag cctacattgt attggcatgt
1141 aaaaaataag cgggctttgc tcgacgcctt agccattgag atgttagata ggcaccatac
1201 tcacttttgc cctttagaag gggaaagctg gcaagatttt ttacgtaata acgctaaaag
1261 ttttagatgt gctttactaa gtcatcgcga tggagcaaaa gtacatttag gtacacggcc
1321 tacagaaaaa cagtatgaaa ctctcgaaaa tcaattagcc tttttatgcc aacaaggttt
1381 ttcactagag aatgcattat atgcactcag cgcagtgggg cattttactt taggttgcgt
1441 attggaagat caagagcatc aagtcgctaa agaagaaagg gaaacaccta ctactgatag
1501 tatgccgcca ttattacgac aagctatcga attatttgat caccaaggtg cagagccagc
1561 cttcttattc ggccttgaat tgatcatatg cggattagaa aaacaactta aatgtgaaag
1621 tgggtcttaa aagcagcata acctttttcc gtgatggtaa cttcactagt ttaaaaggat
1681 ctaggtgaag atcctttttg ataatctcat gaccaaaatc ccttaacgtg agttttcgtt
1741 ccactgagcg tcagaccccg tagaaaagat caaaggatct tcttgagatc cttttttttct
1801 gcgcgtaatc tgctgcttgc aaacaaaaaa accaccgcta ccagcggtgg tttgtttgcc
1861 ggatcaagag ctaccaactc tttttccgaa ggtaactggc ttcagcagag cgcagatacc
1921 aaatactgtt cttctagtgt agccgtagtt aggccaccac ttcaagaact ctgtagcacc
1981 gcctacatac ctcgctctgc taatcctgtt accagtggct gctgccagtg gcgataagtc
2041 gtgtcttacc gggttggact caagacgata gttaccggat aaggcgcagc ggtcgggctg
2101 aacggggggt tcgtgcacac agcccagctt ggagcgaacg acctacaccg aactgagata
2161 cctacagcgt gagctatgag aaagcgccac gcttcccgaa gggagaaagg cggacaggta
2221 tccggtaagc ggcagggtcg gaacaggaga gcgcacgagg gagcttccag ggggaaacgc
2281 ctggtatctt tatagtcctg tcgggtttcg ccacctctga cttgagcgtc gatttttgtg
2341 atgctcgtca ggggggcgga gcctatggaa aaacgccagc aacgcggcct ttttacggtt
2401 cctggccttt tgctggcctt ttgctcacat gacccgacac catcgaatgg tccctatcag
2461 tgatagagat tgacatccct atcagtgata gagatactga gcacatcagc aactttaaga
2521 aggagatcgg ccgatggata ttaatactga aactgagatc aagcaaaagc attcactaac
2581 ccccttttcct gttttcctaa tcagcccggc atttcgcggg cgatattttc acagctattt
2641 caggagttca gccatgaacg cttattacat tcaggatcgt cttgaggctc agagctgggc
2701 gcgtcactac cagcagctcg cccgtgaaga gaaagaggca gaactggcag acgacatgga
2761 aaaaggcctg ccccagcacc tgtttgaatc gctatgcatc gatcatttgc aacgccacgg
2821 ggccagcaaa aaatccatta cccgtgcgtt tgatgacgat gttgagtttc aggagcgcat
2881 ggcagaacac atccggtaca tggttgaaac cattgctcac caccaggttg atattgattc
2941 agaggtataa aacgaatgag tactgcactc gcaacgctgg ctgggaagct ggctgaacgt
3001 gtcggcatgg attctgtcga cccacaggaa ctgatcacca ctcttcgcca gacggcattt
3061 aaaggtgatg ccagcgatgc gcagttcatc gcattactga tcgttgccaa ccagtacggc
3121 cttaatccgt ggacgaaaga aatttacgcc tttcctgata agcagaatgg catcgttccg
3181 gtggtgggcg ttgatggctg gtcccgcatc atcaatgaaa accagcagtt tgatggcatg
```

3241 gactttgagc aggacaatga atcctgtaca tgccggattt accgcaagga ccgtaatcat
3301 ccgatctgcg ttaccgaatg gatggatgaa tgccgccgcg aaccattcaa aactcgcgaa
3361 ggcagagaaa tcacggggcc gtggcagtcg catcccaaac ggatgttacg tcataaagcc
3421 atgattcagt gtgcccgtct ggccttcgga tttgctggta tctatgacaa ggatgaagcc
3481 gagcgcattg tcgaaaatac tgcatacact gcagaacgtc agccggaacg cgacatcact
3541 ccggttaacg atgaaaccat gcaggagatt aacactctgc tgatcgccct ggataaaaca
3601 tgggatgacg acttattgcc gctctgttcc cagatatttc gccgcgacat tcgtgcatcg
3661 tcagaactga cacaggccga agcagtaaaa gctcttggat tcctgaaaca gaaagccgca
3721 gagcagaagg tggcagcatg acaccggaca ttatcctgca gcgtaccggg atcgatgtga
3781 gagctgtcga acaggggat gatgcgtggc acaaattacg gctcggcgtc atcaccgctt
3841 cagaagttca caacgtgata gcaaaacccc gctccggaaa gaagtggcct gacatgaaaa
3901 tgtcctactt ccacaccctg cttgctgagg tttgcaccgg tgtggctccg gaagttaacg
3961 ctaaagcact ggcctgggga aaacagtacg agaacgacgc cagaaccctg tttgaattca
4021 cttccggcgt gaatgttact gaatccccga tcatctatcg cgacgaaagt atgcgtaccg
4081 cctgctctcc cgatggttta tgcagtgacg gcaacggcct tgaactgaaa tgcccgttta
4141 cctcccggga tttcatgaag ttccggctcg gtggtttcga ggccataaag tcagcttaca
4201 tggcccaggt gcagtacagc atgtgggtga cgcgaaaaaa tgcctggtac tttgccaact
4261 atgacccgcg tatgaagcgt gaaggcctgc attatgtcgt gattgagcgg gatgaaaagt
4321 acatggcgag ttttgacgag atcgtgccgg agttcatcga aaaaatggac gaggcactgg
4381 ctgaaattgg ttttgtattt ggggagcaat ggcgatgacg catcctcacg ataatatccg
4441 ggtaggcgca atcactttcg tctactccgt tacaaagcga ggctgggtat ttcccggcct
4501 ttctgttatc cgaaatccac tgaaagcaca gcggctggct gaggagataa ataataaacg
4561 aggggctgta tgcacaaagc atcttctgtt gagttaagaa cgagtatcga gatggcacat
4621 agccttgctc aaattggaat caggtttgtg ccaataccag tagaaacaga cgaagaatcc
4681 atgtcttaat agagatactt cgcatcatgt gttccggagt ttctttgtcc tcctataacg
4741 aaaatcttct acaacagctt tttgaacttt ttaagcaaaa gagctgatcc tccgtcagct
4801 catatatata tatctattat atatatatat ttagggattt gatttcacga gagagatttg
4861 caactcttgg tggtagactt tgcaactctt ggtggtagac tttgcaactc ttggtggtag
4921 actttgcaac tcttggtggt agacttggtc ataatggact tttgttaaaa aatttcttaa
4981 aatcttagag ctccgatttt gaatagcttt ggttaagaaa atgggctcga tggctttcca
5041 taaaagtaga ttgtttttaa cttttgggga cgcgtcggaa atttggttat ctactttatc
5101 ttatctaact agaaaaaatt atgcgtctgg gattaacttt cttgtttctt tagagattct
5161 ggatttatcg gaaaccttga taaaggctat ttctcttgac cacagcgaat ctttgtttaa
5221 aatcaagtct ctagatgttt ttaatggaaa agttgtttca gaggcatcta aacaggctag
5281 agcggcatg
