# Supplementary figures and images for: Development of a lambda Red based system for gene deletion in Chlamydia
Source: PLoS One. 2024 Nov 14;19(11):e0311630. doi: 10.1371/journal.pone.0311630 (PMC11563418; doi:10.1371/journal.pone.0311630)

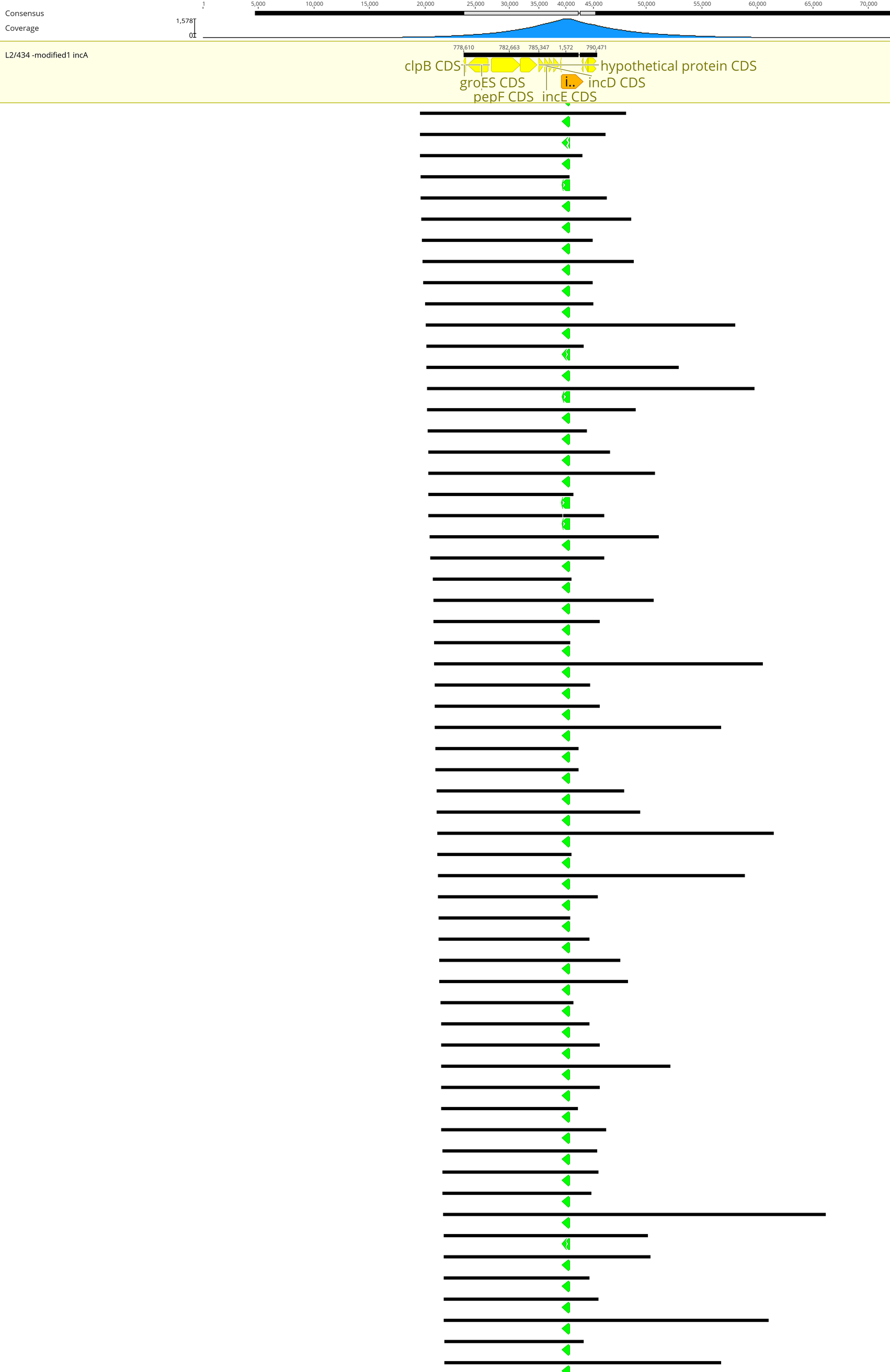

Supplement: S2 Fig — Read map alignment of ~1500 long-read sequences mapped onto the reference genome of C. trachomatis L2/434, with ~95% exhibiting a Q-score of at least Q30 (1:1000 base call error). Each track represents an individual sequencing read, with gaps in alignment suggesting possible structural variations or sequencing artifacts. Highlighted in green are CAT markers. The consistent mapping across the orange highlighted region containing the deleted incA allele suggests a stable recombination event at this locus. (TIF) [file pone.0311630.s003.tif]

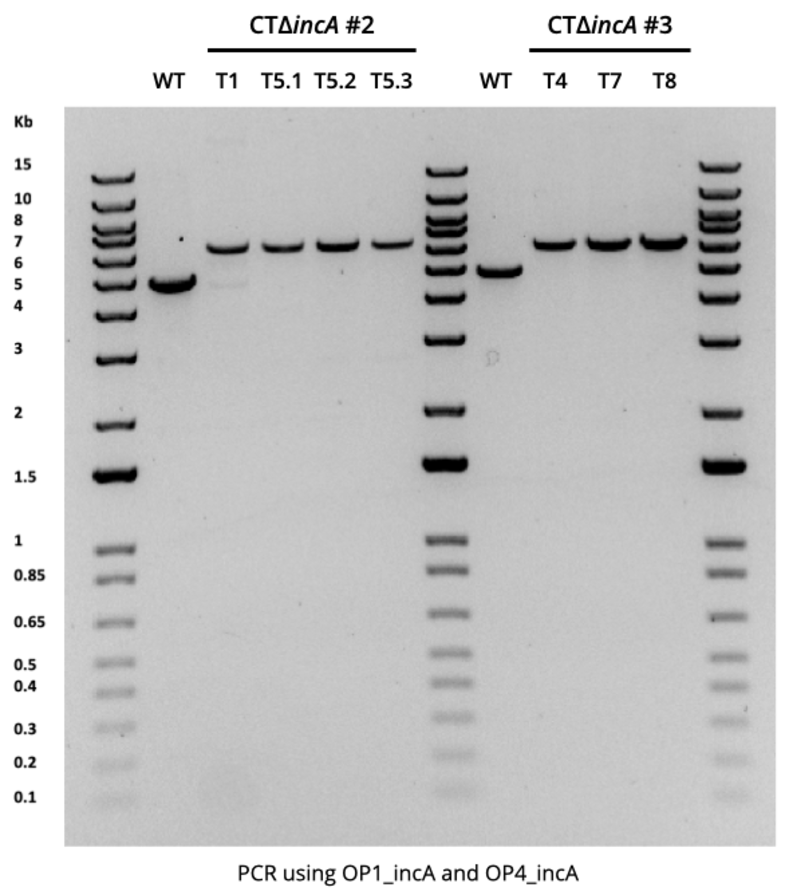

Supplement: S3 Fig — gDNA was isolated from WT C. trachomatis L2 and varying temporal isolates (T1-T8) of the two clones indicated and analyzed by PCR using primers located outside the upstream and downstream ~2 kb flanking sequences. Lower weight bands containing the WT incA gene were abundant only in gDNA from WT C. trachomatis. Higher weight amplicons containing the larger gfp-cat insert were present only in mutant gDNA, and were detected from all isolates. (TIF) [file pone.0311630.s004.tif]
