# Supplementary material for: Development of a lambda Red based system for gene deletion in Chlamydia
Source: PLoS One. 2024 Nov 14;19(11):e0311630. doi: 10.1371/journal.pone.0311630 (PMC11563418; doi:10.1371/journal.pone.0311630)
Supplement: S1 Raw images — (PDF) [file pone.0311630.s005.pdf]

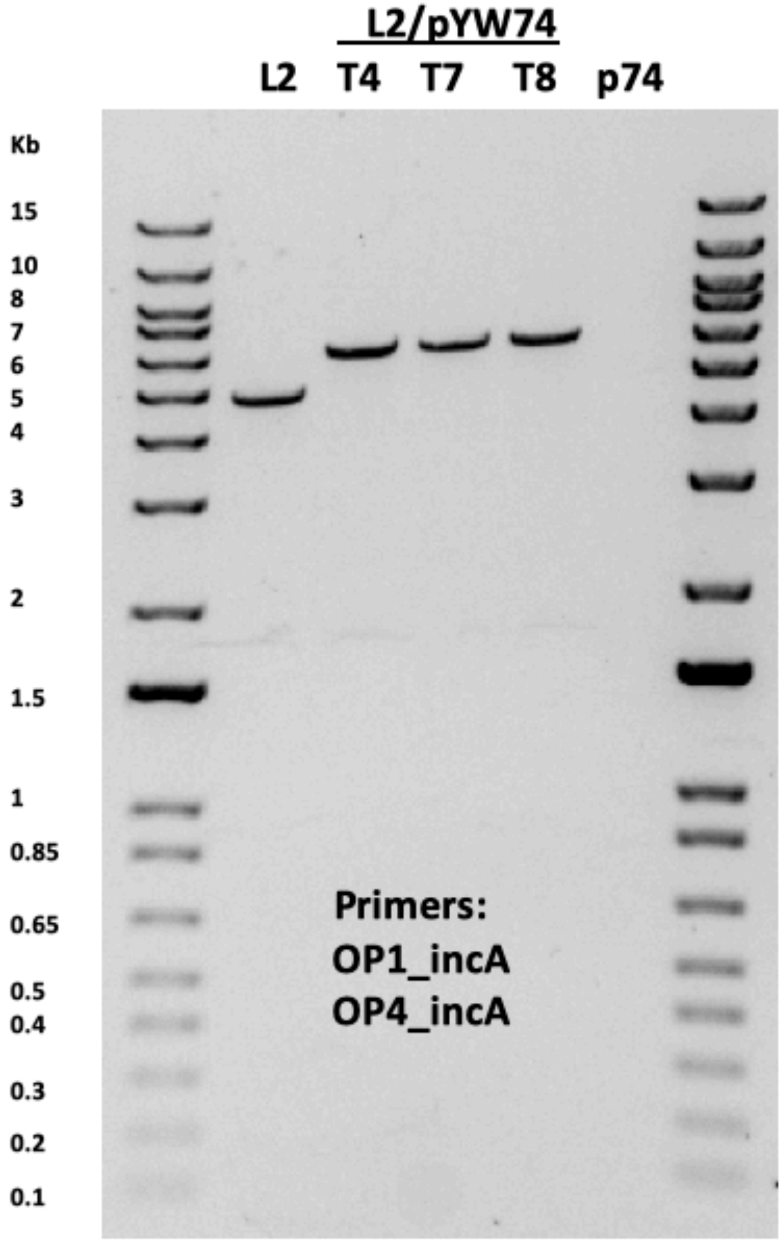

PCR using Q5, annealing at 65oC 5", extension @72oC 3'10", final for 5min. Use 1ul PCR on 0.9% gel, 100 V for 120min. Use 1kb plus as DNA ladder.

Expected sizes of PCR products using primers OP1_incA and OP4_incA:

L2 wild type, 4.9 kb
L2/pYW74 (incA deletion), 6.1kb

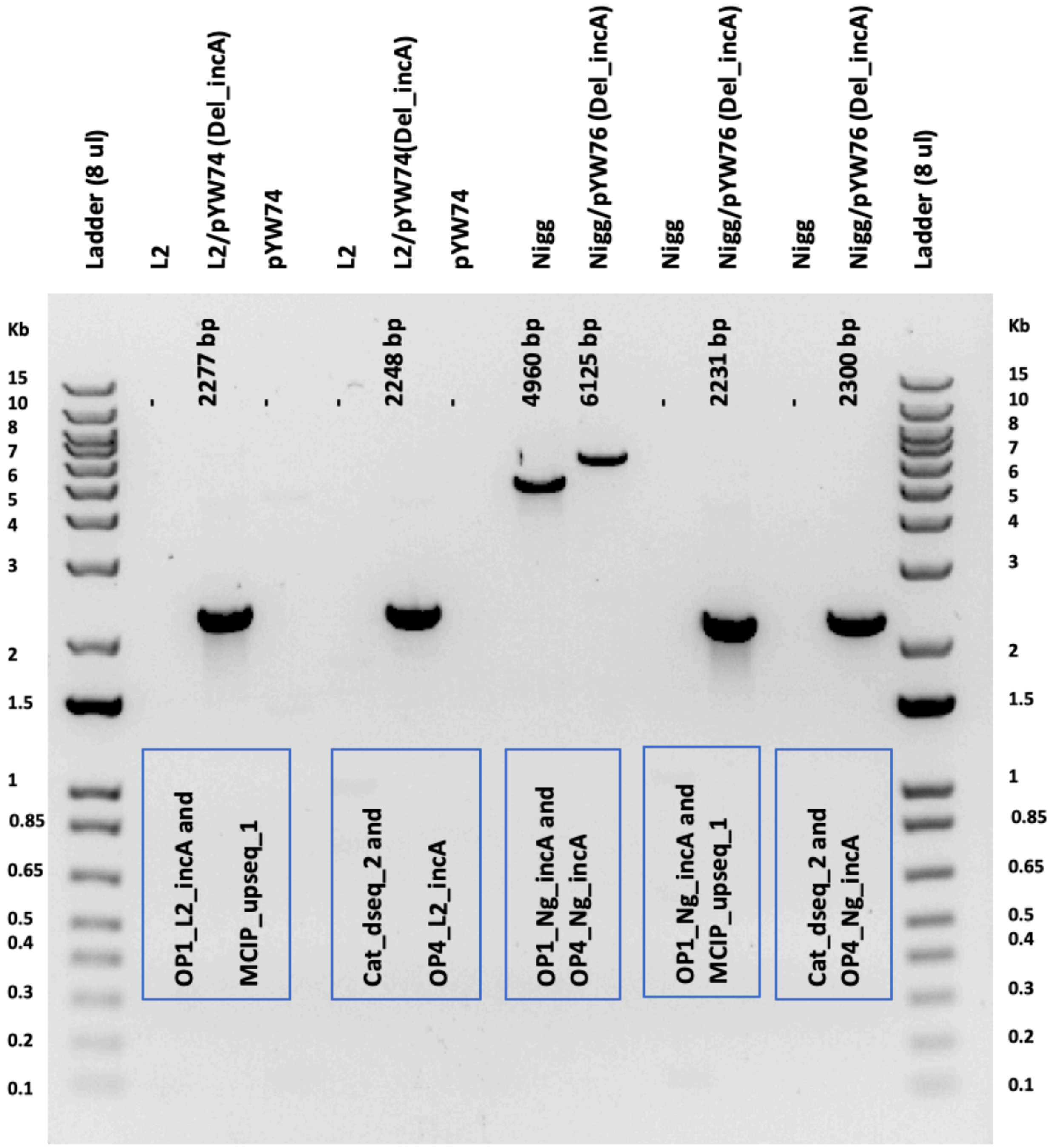

Gel_20240501

PCR using Q5, annealing at 62oC 5”, extension @72oC 3’10”, 35 cycles, final 72oC for 5min.
Use 2ul PCR product on 0.9% gel, 100 volts for 120 min. L= 1kb+ DNA ladder (Invitrogen)

All PCR products are as expected.

Gel_20230321 PCR with OP1/OP4 in L2/pYW85 and L2/pYW89

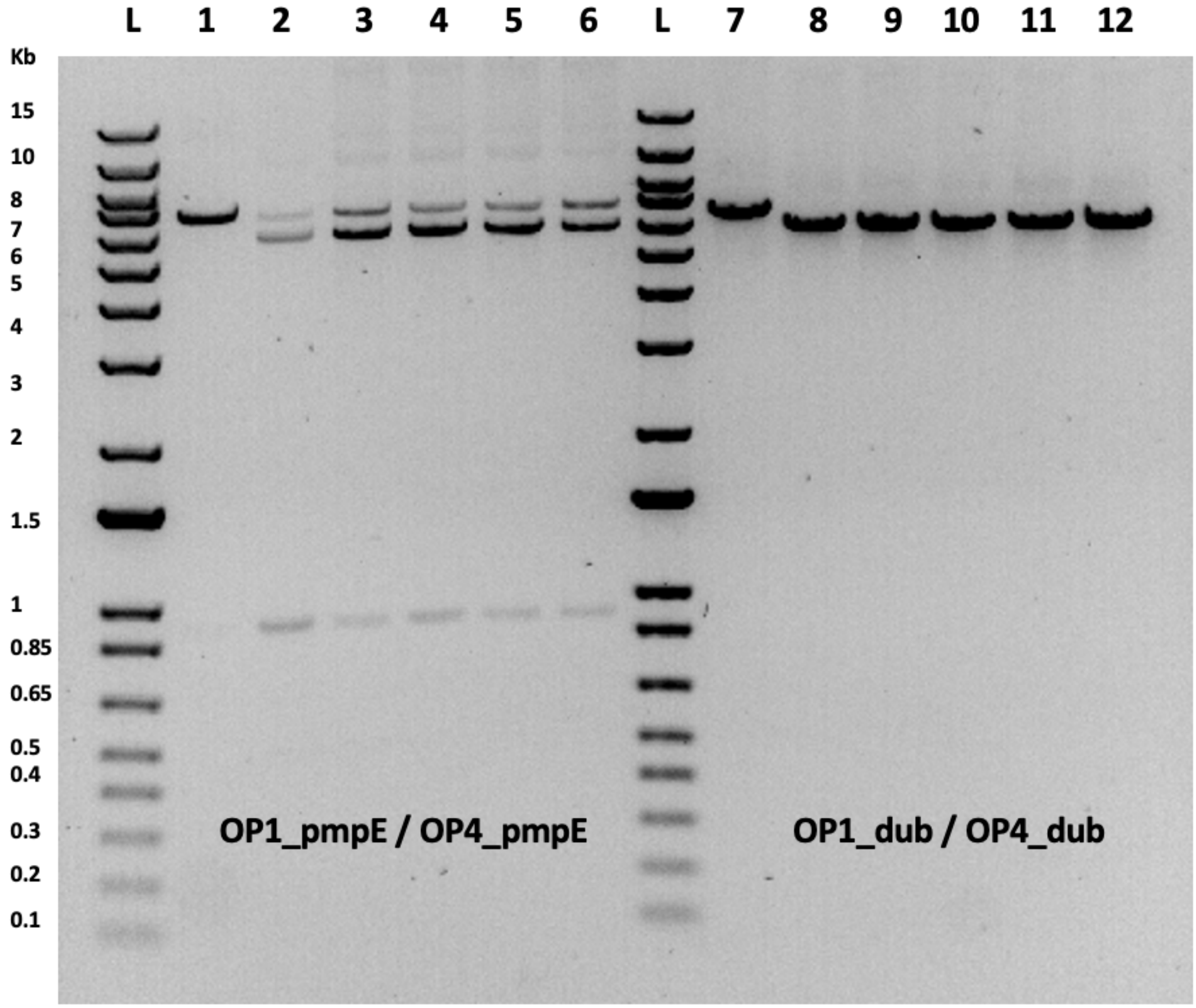

25ul PCR using Q5, annealing at 64oC 5”, extension @72oC 4’, final for 7min.
2ul PCR + 2ul purple dye (6x) + 6ul H2O. Use 5ul on 0.9% gel, 100 V for 120min.
Use 5ul 1kb plus DNA ladder as PCR product size reference.

Primers: Lane 1-6: OP1_pmpE / OP4_pmpE; Lane 7-12: OP1_dub / OP4_dub

DNA templates (gDNA)
Lane 1: L2 wt (7 kb)
Lane 2: L2/pYW85 (T2) (pmpE deletion) (6 kb)
Lanes 3-6: L2/pYW85 (T5 clones No. 1-4)
Lane 7: L2 wt (6.5 kb)
Lane 8: L2/pYW89 (T2) (dub1&2 deletion) (6 kb)
Lanes 9-12: L2/pYW89 (T5 clones No. 5-8)

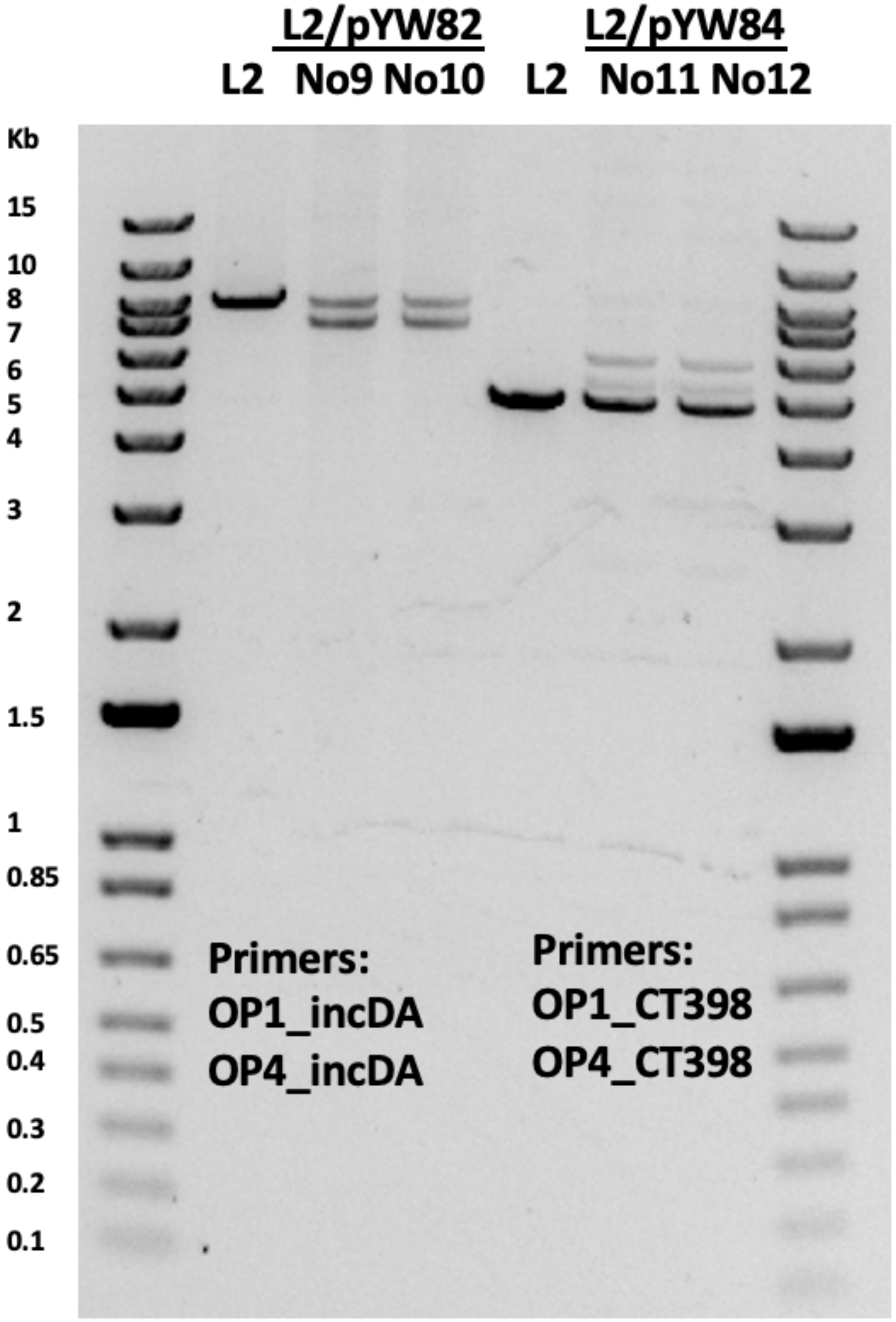

PCR using Q5, annealing at 63oC 5", extension @72oC 4', final for 7min.
Use 1ul PCR on 0.9% gel, 100 V for 120min.
Use 1kb plus as DNA ladder.
